# The impact of summer programming on the obesogenic behaviors of children: behavioral outcomes from a quasi-experimental pilot trial

**DOI:** 10.1186/s40814-020-00617-x

**Published:** 2020-05-28

**Authors:** R. Dugger, K. Brazendale, E. T. Hunt, J. B. Moore, G. Turner-McGrievy, K. Vogler, M. W. Beets, B. Armstrong, R. G. Weaver

**Affiliations:** 1 Department of Exercise Science, University of South Carolina, Columbia, South Carolina USA; 2 Department of Health Sciences, University of Central Florida, Orlando, Florida USA; 3 Department of Implementation Science, Wake Forest School of Medicine, Winston-Salem, North Carolina USA; 4 Department of Health Promotion, Education, and Behavior, University of South Carolina, Columbia, South Carolina USA; 5 Department of Instruction and Teacher Education, University of South Carolina, Columbia, South Carolina USA

**Keywords:** Structure, Intervention, Physical Activity, Diet, Sleep, Screen time

## Abstract

**Background:**

Children from low-income families experience accelerated BMI gain and learning loss during summer. Healthy Summer Learners (HSL) addresses accelerated BMI gain and academic learning loss during summer by providing academic- and health-focused programming. This manuscript reports the effects of HSL on underlying obesogenic behaviors (i.e., physical activity, screen time, sleep, diet) that lead to accelerated summer BMI gain, a necessary first step to informing a future randomized controlled trial of HSL.

**Methods:**

In the summer of 2018 and 2019 using a quasi-experimental study design, 180 children (90 per summer, 7.9 years [SD = 1.0], 94% non-Hispanic Black, 40% male) at two schools (i.e., one per summer) who were struggling academically (25–75% on a standardized reading test) were provided a free, school-based 6-week health- and academic-focused summer program (i.e., HSL, *n* = 60), a 4- to 6-week academic-focused summer program (i.e., 21st Century Summer Learning program (21C), *n* = 60), or no summer program (*n* = 60). Children wore the Fitbit Charge 2™ over a 10-week period during the summers (June–Aug) of 2018–2019. Differences within (within child days attend vs. not attend) and between (differences between groups attend vs. not attend) were evaluated using mixed effects linear regression.

**Results:**

Regression estimates indicated that, on days attending, HSL children experienced a greater reduction in sedentary minutes (− 58.6 [95% CI = − 92.7, − 24.4]) and a greater increase in moderate-to-vigorous physical activity (MVPA) (36.2 [95% CI = 25.1, 47.3]) and steps (2799.2 [95% CI = 2114.2, 3484.2]) compared to 21C children. However, both HSL and 21C children were more active (i.e., greater MVPA, total steps) and less sedentary (i.e., less sedentary minutes and total screen time) and displayed better sleeping patterns (i.e., earlier and less variability in sleep onset and offset) on days they attended than children in the control.

**Conclusions:**

HSL produced greater changes in physical activity than 21C. However, attendance at either HSL or 21C leads to more healthy obesogenic behaviors. Based on the behavioral data in this pilot study, a larger trial may be warranted. These results must be considered along with the pending primary outcomes (i.e., academics and BMI *z*-score) of the HSL pilot to determine if a full-scale trial is warranted.

**Trial registration:**

NIH-NCT03321071. Registered 25 October 2017

## Background

The months of summer (i.e., June–August) have been identified as a “double jeopardy” of vulnerability for children (5–12 years) from low-income households in the USA, in terms of both health and academic achievement. Over 30 years of empirical evidence indicates that low-income, minority children perform worse on standardized tests than their same-age middle-to-upper-income peers. This achievement gap is attributed almost entirely to declines in academic proficiency experienced by low-income children during the summer [1–4]. At the same time, children’s body mass index (BMI) gain accelerates during summer [5–11]. Moreover, this excessive summer BMI gain is more pronounced for minority children [6, 8, 11], who are more likely to come from socio-economically disadvantaged families [12, 13]. Recently, our research team along with a local school district designed Healthy Summer Learners (HSL) to address both declines in academic proficiency and accelerated BMI gain in one summer program. The component of HSL targeting accelerated summer BMI gain is based on the structured-days hypothesis (SDH).

The SDH suggests that the presence of structure, defined as a pre-planned, segmented, and adult-supervised environment, may regulate children’s engagement in obesogenic behaviors and can lead to maintenance of BMI. For instance, during the school year, children spend the majority of the day in a structured environment (i.e., school) which may beneficially impact their obesogenic behaviors and in turn BMI [14]. However, during the summer when children no longer have access to school, their behaviors may deteriorate and corresponding unhealthy changes in BMI may occur. Evidence for the SDH is largely based upon studies which compared weekdays (i.e., more structured because children attend school) vs. weekend days (i.e., no school and less structured) [14]. In fact, a recent systematic review identified 190 studies that reported weekday (structured days) and weekend (unstructured days) obesogenic behaviors in children during the school year [14]. Overall, 155 studies (82%) showed that children exhibited more healthful behaviors during school weekdays when compared to weekends. Specifically, the SDH posits that the structure provided by the school day would impact children’s physical activity, diet, sleep, and screen time in a positive way. In terms of physical activity and screen time, the SDH posits that the structured day limits children’s engagement in sedentary behaviors, such as watching TV, playing video games, or engaging with mobile screen devices (tablets, cellphones). Further, schools provide planned (e.g., physical education, recess) and incidental (e.g., transitions between lessons/classes) opportunities for children to be physically active. The SDH also posits that children’s sleeping patterns are regulated by the engagement in structured days. Specifically, during the school year, children must arrive at school at a specific time. Thus, children’s bed and wake times are earlier and less variable than they would be on unstructured days. Finally, the SDH posits that on days with greater amounts of structure, such as school days, that the healthfulness of the foods available is regulated. The foods served within schools are typically regulated by federal, state, and/or organizational standards that dictate the nutritional composition and quantity of foods served. Conversely, on unstructured days, such as weekend days during the school year, children are exposed to environments, commonly the home, where less oversight occurs, and children are able to select and consume less healthful foods in larger quantities.

Similar to weekend days during the school year, summer vacation represents a window of vulnerability for many children as the structure that the school day provides is removed. While few studies have examined children’s obesogenic behaviors during the school year compared to the summer, those that have are consistent with the SDH [15–18]. One intervention strategy to mitigate increased engagement in unhealthy behaviors is to provide children with access to structured programming during the summer. We are aware of only one study that has explored the impact of this approach on children’s obesogenic behaviors [19]. This study found that on days that children attended a structured academic-focused summer program they engaged in healthier behaviors. For instance, on days when children attended the program, they participated in approximately 11.3 additional minutes of moderate-to-vigorous physical activity (MVPA) and 76.1 fewer minutes of screen time compared to days they did not attend. However, this study was limited because the program was not designed to impact children’s obesogenic behaviors, it did not include a control group, and it had a small sample (*n* = 30). These limitations make it challenging to estimate expected effects of a structured program targeting health behaviors during the summer.

Although data demonstrates that academic-focused summer programming can positively influence obesogenic behaviors, it is not clear if a health-focused summer program would produce additional benefits than a structured academic program on children’s obesogenic behaviors. Additionally, because evidence for the SDH is primarily based on studies that compare school days to weekend days, it is imperative to explicitly test if this theory can be generalized to summer programming. Thus, the objectives of this manuscript are twofold; the first objective is to assess the impact of providing access to the novel HSL program on the obesogenic behaviors of children compared to a pre-existing academic only program (i.e., 21st Century Summer Learning program [21C]). The second objective is to assess the impact of providing access to HSL and 21C on children’s obesogenic behaviors compared to no programming. Testing if summer programming impacts the presumed behavioral mechanisms underlying accelerated summer BMI gain is a crucial step in order to understand the impact of structure during summer and to guide optimization of future intervention programs. Furthermore, this will allow for the estimation of resources and the size of the sample required for a planned, well-powered randomized controlled trial to evaluate the impact of HSL.

Based on the SDH, we hypothesize that:

Primary hypotheses

- *Hypothesis 1*. On days when they attend a program, children enrolled in a summer program (HSL or 21C) will engage in more beneficial levels of obesogenic behaviors compared to children in the control group.
- *Hypothesis 2*. HSL will impact physical activity and dietary outcomes to a greater degree than the 21C program, while the impact on screen time and sleep behaviors will be similar between programs.

Secondary hypotheses

- *Hypothesis 3*. On days when they attend a program, children will engage in more beneficial levels of obesogenic behaviors compared to weekdays that they do not attend.
- *Hypothesis 4*. On days when they attend a program, children will engage in more beneficial levels of obesogenic behaviors compared to the weekend.
- *Hypothesis 5*. On days when children do not attend a program, all children will engage in similar obesogenic behaviors compared to weekend days.
- *Hypothesis 6*. On days when children in HSL or 21C groups do not attend a summer program, they will engage in similar obesogenic behaviors compared to controls.
- *Hypothesis 7*. There will be no group differences in obesogenic behaviors on weekend days between HSL, 21C, and no program.

## Methods

### Trial design

All procedures were approved by the lead author’s university institutional review board. This study was a three-armed quasi-experimental study employing a repeated measure within and between participant design comparing the HSL (*n* = 60) to 21C (*n* = 60) and no program (*n* = 60). HSL was designed to mitigate academic learning loss and accelerated summer BMI gain by positively impacting children’s obesogenic behaviors. This paper presents the obesogenic behavior outcomes from this pilot trial.

### Participants

To distribute the costs of operating HSL, the study took place over two summers (i.e., June–August, 2018 and 2019). A single elementary school in the Columbia, SC metropolitan area, participated each summer (i.e., two schools, one each summer). Schools were selected because they served children from low-income households (100% free and reduced priced lunch) and they operated a 21C summer program. Inclusion criteria were that children were in the 2nd, 3rd, or 4th grade and their standardized reading score on the Measures of Academic Progress was between the 25th and the 75th percentile. Exclusion criteria was the presence of a severe intellectual disability (e.g., fragile X syndrome, severe autism). Measures of Academic Progress scores between the 25th and the 75th percentile and the absence of a severe intellectual disability were chosen as inclusion/exclusion criteria because these are the criteria the school district used to select children to participate in the 21C. A total of 90 students participated each summer. A total of 1281 student records were assessed for eligibility (*n* = 617 in 2018 and *n* = 664 in 2019) via school records. Of these 1281 students, 813 were not eligible to participate because of their MAP reading scores (*n* = 173) or they were not in the 2nd–4th grade (*n* = 640). The 408 eligible children were recruited to participate in the study via informational fliers and consent forms sent home from school. A total of 269 families declined for their child to participate and 199 returned a signed consent form. A total of 180 children were randomly selected to participate in the study. Of the 180 children, 60 were enrolled in the 21C program (i.e., *n* = 30 in 2018 and *n* = 30 in 2019). The remaining 120 (i.e., *n* = 60 in 2018 and *n* = 60 in 2019) were randomly allocated to one of two conditions HSL or control (no program) using a random number generator.

### Interventions

HSL was designed in partnership with the local school district in which the study occurred. HSL was designed to (1) address summer declines in reading achievement and (2) mitigate accelerated unhealthy BMI gain during the summer by positively impacting children’s obesogenic behaviors. HSL operated at the participating schools and was delivered by certified teachers. Participants enrolled in HSL attended daily (Monday–Thursday, 8:00–15:30) for 6 weeks during the summer, with a 1-week break in the middle of the program (i.e., 4th of July). The program day consisted of alternating 60-min periods of physical activity (3 total hours) with 1 h and 45-min periods of reading instruction (3.25 total hours). All participants were provided a United States Department of Agriculture (USDA) Summer Feeding Program compliant breakfast (8:00–8:30), lunch (12:30–13:00), and a healthy snack. The USDA Summer Feeding Program mandates that foods align with the meal patterns laid out by this program. For breakfast, this includes the provision of both fruits and vegetables and whole grain options and excluding sugar-sweetened milk beverages. In addition, breakfasts cannot exceed 500 kcal, 10% kcal from saturated fat, and 430 mg of sodium, and lunches will not exceed 650 kcal, 10% kcal from saturated fat, and 640 mg of sodium. Lunches are required to include milk, fruits and/or vegetables, a grain, and a meat or meat alternative. For snacks, the program could choose two of the four components of the lunch guidelines and had to include a fruit or vegetable. A 15-min nutrition education session, based on the USDA Team Nutrition Curriculum [20], was delivered by one of the teachers each day during lunch. The USDA Team Nutrition Curriculum focuses on eating a variety of low-fat foods and incorporating fruits, vegetables, and whole grains into children’s diets daily. Nutrition Education sessions typically consisted of a 5-min lesson by a certified teacher followed by a 10-min activity session (e.g., activity sheets, comic book readings).

The 21C is a federally funded program providing academic enrichment opportunities for students who attend low-performing schools. Students enrolled in 21C attended the same school as children enrolled in HSL. The 21C operated daily (Monday–Thursday) from 8:30–14:00 for 4 weeks during the summer of 2018 and 6 weeks during the summer in 2019 at the participating schools. The program day consisted of academic sessions in the morning and afternoon (9:00–11:30 and 12:30–13:45) and 1 h of physical activity before lunch (11:30–12:30). All participants were provided a USDA Summer Feeding Program compliant breakfast (8:30–9:00) and lunch (11:30–12:30). Children in the control group did not attend either program.

### Outcomes

#### Physical activity and sleep

Physical activity (steps, MVPA, sedentary time) and sleep (total sleep duration, sleep onset, and sleep offset) were measured using a Fitbit Charge 2^TM^ (Fitbit Inc., San Francisco, CA, USA). Fitbit Charge 2^TM^ devices have demonstrated initial reliability and validity for detecting heartrate and sleep [21–23]. Each Fitbit was assigned a unique numeric identifier, and each device was linked to Fitabase (San Diego, CA, USA), a web-based interface that allows remote access and download of participants’ second-level Fitbit data. Children were asked to wear the Fitbit device every day for the entire summer (i.e., 12 weeks) starting the last week of school in the spring (May) until the first week of school in fall (August). Parents were sent text reminders to sync and charge their child’s Fitbit device every 3–4 days. Only days with >10 h of waking wear [24] and with step estimates between 1000 and 30,000 steps [25, 26] were considered valid. Data processing was informed by the ISCOLE data processing protocols [24].

Consistent with previous studies [27–30], daily resting heartrate for each child was distilled into activity intensity levels by identifying the average of the lowest 10-min beats-per-minute during wake time for each day. Resting heartrates that were above the 95th (90 bpm) or below the 5th (50 bpm) percentile were considered implausible and replaced with the closest day that the child had a plausible resting heartrate. Percent heart rate reserve (HRR) was calculated using the following formula: $\frac{\mathrm{heart}\ \mathrm{rate}-\mathrm{resting}\ \mathrm{heart}\ \mathrm{rate}}{\mathrm{maximum}\ \mathrm{heart}\ \mathrm{rate}-\mathrm{resting}\ \mathrm{heart}\ \mathrm{rate}}$ and was used to determine activity intensity levels, with 0.0–19.9% of HRR considered sedentary, 20.0–49.9% of HRR considered light physical activity, and ≥ 50.0% considered MVPA [28, 29]. Maximum heart rate was defined as 197 beats per minute for all children [31]. 

Sleep was identified and parsed from physical activity. Sleep onset was defined as the first minute that the sleep episode began. Sleep offset was selected as the last minute that a sleep episode was recorded. The sum of the minutes that the Fitbit device classified a child as asleep during a sleep episode was considered total sleep time. Consistent with past research [32], the variability in sleep onset and offset was represented by calculating the standard deviation in sleep onset and offset for each child. Only nocturnal sleep was considered for this study and was defined as sleep onset that occurred between 5 pm and 6 am and lasted for greater than 240 min [33]. Sleep segments separated by less than 20 min were considered one continuous sleep segment.

#### Screen time and diet

Parents completed the following measures through an online survey that was texted to their smartphone twice per week (i.e., one weekday and one weekend day) during the 12-week period that children wore the Fitbit device and were encouraged to complete these measures with their children to improve accuracy.

Parents were asked to estimate the total amount of time (hours and minutes) their child spent in front of a screen that day (e.g., TV, computer, video game, smartphone, and tablet) and the total amount of time spent using screens after 20:00 h [34, 35]. Parents reported on screen time on both weekend and weekdays during the program period (HSL = 6 weeks, 21C = 4 weeks).

Diet was assessed via a parent report food screener [36]. Items were scored by four possible response categories consiting of the following: 0 (child did not consume), 1 (child consumed a little), 2 (child consumed some), and 3 (child consumed a lot). Individual food items were grouped in accordance with the Healthy Meal Index (HMI) [37]. Food categories included fruits, vegetables, dairy (non-sugar based), convenience foods, sweets and desserts, and sugar-sweetened beverages. Consumption was dichotomized (i.e., “did” vs. “did not” consume) and reported as mean days/week [36]. Two variables were created for analysis of diet: healthy foods/drinks (fruits, vegetables, and dairy) and unhealthy foods/drinks (convenience foods, and sweets/desserts, sugar-sweetened beverages).

### Sample size

Given the focus on hypothesis testing for this study, it is essential to show the study is sufficiently powered to detect differences when they exist between intervention groups [38, 39]. The above selection criteria yielded 60 children in each intervention arm. According to the statistical software G*power 3.1.9.7, the study is sufficiently powered to detect a difference between intervention groups of *d* = 0.18 with a power = 80% and *α* = 0.05. This is true for each obesogenic behavior outcome and all hypotheses tested. The only previous study of which we are aware that has examined the impact of structured summer programming on obesogenic behaviors found Cohen’s *d* effects of 0.21 (i.e., diet) to 0.78 (i.e., physical activity) [19]. Based on this initial data, the current study was determined to have adequate power to detect a significant difference, should one exist.

### Randomization

Random assignment was completed by the last author (RGW) who was not involved in data collection and was completed each summer after participants enrolled in the study using the runiform command in Stata (v14.2, College Station, TX). Once implemented, randomization could not be changed.

### Data analysis

All analyses were completed in Stata (v14.2, College Station, TX). Descriptive statistics of program and child characteristics and outcome variables were examined. Because observations were nested within children, violating the assumption of independence, mixed effects linear regressions with days nested within children were estimated. A single model to test all hypotheses was estimated for each obesogenic behavior (i.e., sleep, sedentary, physical activity, diet). Each model examined between program differences in behaviors (e.g., HSL vs. control) and the difference in behavior change between programs (e.g., HSL vs. 21C) by type of day (hypotheses 1 and 2). Additionally, within-participant differences in obesogenic behaviors on weekdays children attended a program, weekdays children did not attend a program, weekend days (hypotheses 3–5), and between-group differences by type of day (hypotheses 6–7) were examined. Each model included type of day (weekday attend program, weekday not attend program, and weekend day), program (HSL, 21C, or control), and program-×-type of day interactions as the independent variables and obesogenic behavior as the dependent variable. All models included age, gender, and year of participation (i.e., summer of 2018 or 2019) as covariates. Robust standard errors were used using the “robust” command in STATA because the Breusch-Pagan test of normality indicated that some of the models violated the assumption of normally distributed residuals.

Consistent with past research [14, 40], and hypothesis testing theory stating that a set of related hypotheses that provide consistent results strengthens causal inference [38], a coding system was adopted to explore the hypotheses. Results that were statistically significant (determined by a 95% confidence interval that did not cross zero) and in the direction of the hypothesized relationship as well as those that were not statistically significantly different when there was a hypothesis suggesting no difference should exist were classified as “Support.” Results that were not statistically significant despite a hypothesis suggesting a mean difference were classified as “Null.” Results that were statistically significant but contrary to the hypothesized relationship were classified as “Conflict.”

## Results

**Fig. 1:**
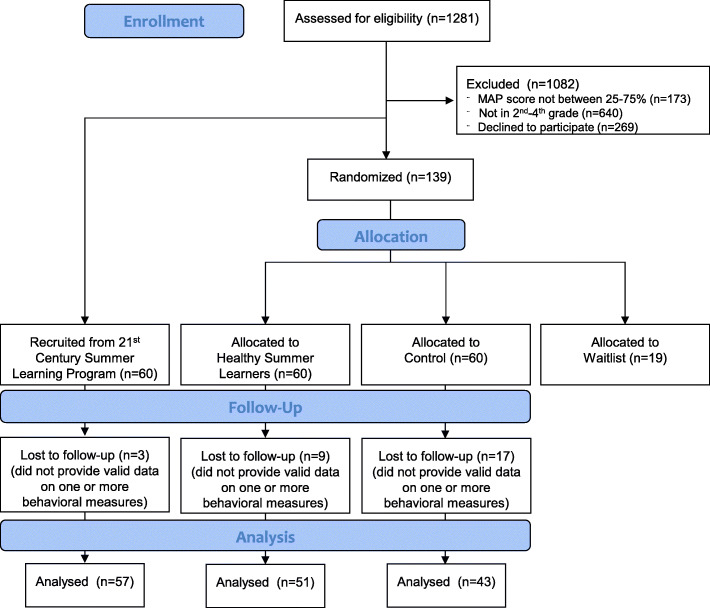
Consort diagram showing the flow of participants through each stage of the study. MAP, Measures of Academic Progress Standardized Reading Test

All recruitment was completed in the Spring (April and May of 2018 and 2019). Demographics of the participating children are presented in Table 1. Raw behavioral estimates by study arm, year or participation, and condition are presented in Supplemental Tables 1-3. Figure 1 presents the flow of participants in the study via the Consort diagram. Children enrolled in the study (*n* = 180) were primarily non-Hispanic black (i.e., 94%), had a mean age of 7.9 years, and were 60% female.

**Table 1:** Demographics of participants by program

Program	No program	Healthy Summer Learners	21st Century Learning Center
Number of participants	60	60	60
Mean age in years	7.9 (1.0)	7.9 (1.0)	8.0 (1.0)
Male (%)	45.3	48.5	27.4
Participants by race (%)			
Non-Hispanic Black	93.8	92.4	93.5
Other	6.2	7.6	6.5
Program operating days (*n*)	0	24	16/24^a^
Median days attended (*n*)	0	18	15/20^a^
Valid days of measure (*n*)			
Physical activity (SD)	28.8 (19.4)	27.1 (18.7)	35.1 (22.6)
Sleep (SD)	19.9 (15.2)	17.0 (16.5)	25.7 (20.0)
Screen Time (SD)	7.5 (5.1)	5.6 (4.1)	8.4 (5.4)
Diet (SD)	7.5 (5.1)	5.7 (4.2)	8.9 (5.3)

*Abbreviations*: SD standard deviation, *MVPA* moderate-to-vigorous physical activity. ^a^During the second summer, the 21st Century Learning Center extends the program from 16 to 24 days and the median attendance was 20 days

### Behavioral outcomes by hypotheses

*Hypothesis 1. On days when they attend a program, children enrolled in a summer program (HSL or 21C) will engage in more beneficial levels of obesogenic behaviors compared to children in the control group.*

Table 2 presents the findings for the differences in behaviors between HSL vs. control and 21C vs control, on days that children attended their respective programs. Consistent with hypothesis 1, children in HSL engaged in more favorable levels of sedentary time, total steps, sleep onset, sleep offset, sleep onset and offset variability, total screen time, and unhealthy foods/drinks on days they attended HSL when compared to control weekdays. Differences in sleep duration, screen time after 20:00 h, and healthy foods/drinks were not statistically significant between children attending HSL and the control group. Consistent with hypothesis 1, children in 21C engaged in more favorable levels of sedentary time, MVPA, total steps, sleep onset, sleep offset, and sleep onset and offset variability on days they attended 21C when compared to control weekdays. There was no statistically significant difference in sleep duration, total screen time, screen time after 20:00 h, healthy foods/drinks, and unhealthy foods/drinks between days children attended 21C and the control group.

**Table 2:** Model estimated between program differences across weekdays attended

	Healthy Summer Learners (*n* = 51) versus control (*n* = 43)				21st Century Learning (*n* = 57) versus control (*n* = 43)			
Hypothesis 1 Weekday attend vs. weekday control	Est	95% CI	HR	HS	Est	95% CI	HR	HS
**Physical activity**								
Sedentary (min)	**− 134.7**	**(− 194.9, − 74.4)**	−	Support	**− 138.3**	**(− 205.9, − 70.6)**	−	Support
MVPA (min)	**54.8**	**(34.7, 75.0)**	+	Support	**22.7**	**(4.5, 40.8)**	+	Support
Total steps	**6533.3**	**(5321.9, 7744.6)**	+	Support	**3532.0**	**(2332.6, 4731.4)**	+	Support
**Sleep**								
Sleep duration (min)	− 13.6	(− 39.9, 12.8)	+	Null	− 13.0	(− 35.5, 9.4)	+	Null
Sleep onset	**− 156.6**	**(− 193.3, − 120.2)**	−	Support	**− 139.7**	**(− 173.3, − 105.6)**	−	Support
I-I SD for sleep onset	**− 97.8**	**(− 142.9, − 52.8)**	−	Support	**− 86.4**	**(− 134.2, − 38.7)**	−	Support
Sleep offset	**− 150.2**	**(− 215.0, − 86.0)**	−	Support	**− 150.5**	**(− 211.7, − 89.8)**	−	Support
I-I SD for sleep offset	**− 99.0**	**(− 129.4, − 68.5)**	−	Support	**− 71.6**	**(− 114.2, − 29.0)**	−	Support
**Screen time**								
Total screen time (min)	**− 64.2**	**(− 112.0, − 16.3)**	−	Support	− 38.6	(− 84.7, 7.5)	−	Null
Screen time after 20:00 h (min)	− 21.0	(− 52.8, 10.8)	−	Null	− 21.0	(− 46.6, 4.7)	−	Null
**Diet**								
Healthy foods/drinks	0.08	(− 0.28, 0.43)	+	Null	− 0.08	(− 0.44, 0.28)	+	Null
Unhealthy foods/drinks	**− 0.80**	**(− 1.45, −0.16)**	−	Support	− 0.39	(− 1.05, 0.26)	−	Null

*Abbreviations*: *HR* hypothesized relationship, *HS* hypothesized support (i.e., did the relationship support the hypothesis), *HSL* Healthy Summer Learners, *21C* 21st Century Summer Learning Center, *I-I* intra-individual; estimates are based on multilevel mixed effects linear regressions with days nested within children, all estimates represent combined data from schools 1 and 2 (i.e., summer 2018 and 2019); bolded values indicated a statistically significant difference at *p* < 0.05. *Support* indicates statistically significant difference in the hypothesized direction or no statistically significant difference when no difference was hypothesized, *Null* indicates no statistically significant difference despite a hypothesis that means should differ, and *Conflict* indicates statistically significant difference that is contrary to the hypothesized relationship

*Hypothesis 2. HSL will impact physical activity and dietary outcomes to a greater degree than the 21C program, while the impact on screen time and sleep behaviors will be similar between programs.*

Table 3 presents the findings for the differential impact of HSL when compared to 21C on children’s obesogenic behavior. Consistent with hypothesis 2, HSL had a greater positive impact on children’s sedentary time, MVPA, and total steps than 21C. However, contrary to the hypothesis, HSL did not have a greater positive impact on children’s consumption of healthy and unhealthy foods/drinks. Sleep duration, sleep onset and offset, sleep onset and offset variability, total screen time, and screen time after 20:00 h were impacted similarly by HSL and 21C.

**Table 3:** Model estimated difference in change of obesogenic behaviors attend vs. not attend on weekdays

Hypothesis 2 Weekday attend	∆ HSL (*n* = 51) versus 21C (*n* = 57) (interaction)	95% CI	HR	HS
Sedentary (min)	**− 58.6**	**(− 104.3, − 12.8)**	−	Support
MVPA (min)	**36.2**	**(16.4, 55.9)**	+	Support
Total steps	**2799.2**	**(1594.4, 4004.0)**	+	Support
Sleep duration (min)	− 4.4	(− 32.3, 23.4)	0	Support
Sleep onset	35.3	(− 4.3, 74.8)	0	Support
I-I SD for sleep onset	− 1.6	(− 50.3, 47.1)	0	Support
Sleep offset	9.3	(− 24.4, 43.1)	0	Support
I-I SD for sleep offset	− 19.4	(− 69.8, 30.9)	0	Support
Total screen time (min)	13.3	(− 29.6, 56.1)	0	Support
Screen time after 20:00 h (min)	21.1	(− 6.3, 48.5)	0	Support
Healthy foods/drinks	0.24	(− 0.14, 0.62)	+	Null
Unhealthy foods/drinks	0.22	(− 0.51, 0.95)	−	Null

*Abbreviations*: *HR* hypothesized relationship, *HS* hypothesized support (i.e., did the relationship support the hypothesis), *HSL* Healthy Summer Learners, *21C* 21st Century Summer Learning Center, *I-I* intra-individual; estimates are based on multilevel mixed effects linear regressions with days nested within children, all estimates represent combined data from schools 1 and 2 (i.e., summer 2018 and 2019); bolded values indicated a statistically significant difference at *p* < 0.05. *Support* indicates statistically significant difference in the hypothesized direction or no statistically significant difference when no difference was hypothesized, *Null* indicates no statistically significant difference despite a hypothesis that the means should differ, and *Conflict* indicates statistically significant difference that is contrary to the hypothesized relationship

*Hypothesis 3. On days when they attend a program, children will engage in more beneficial levels of obesogenic behaviors compared to weekdays that they do not attend.*

Table 4 presents the results for hypothesis 3. In HSL, consistent with the hypothesis, sedentary time, MVPA, total steps, sleep onset and offset, sleep onset and offset variability, total screen time, and unhealthy foods/drinks were more favorable on days a child attended compared to weekdays they did not attend. However, there was not a statistically significant difference between days attending and weekdays not attending for sleep duration, total screen time after 20:00 h, and healthy foods/drinks. Contrary to the hypothesis, total sleep time decreased on days that children attended HSL compared to weekdays they did not attend. In 21C, consistent with the hypothesis, sedentary time, MVPA, total steps, sleep onset and offset, sleep onset and offset variability, total screen time, total screen time after 20:00 h, and unhealthy foods/drinks were more favorable on days a child attended compared to weekdays they did not attend. However, there was no statistically significant difference between days attending and weekdays not attending for healthy foods/drinks, and contrary to the hypothesis, total sleep time decreased on days that children attended 21C compared to weekdays they did not attend.

**Table 4:** Model estimated within program differences across weekdays attended, weekdays not attended, and weekend days

		Hypothesis 3						Hypothesis 4						Hypothesis 5			
Group	Behaviors	Weekday attend versus weekday not attend			HR	HS		Weekday attend versus weekend day			HR	HS		Weekday not attend versus weekend day		HR	HS
		Est	95% CI					Est	95% CI					Est	95% CI		
HSL (*n* = 51)	Sedentary (min)	**− 138.8**	**(− 171.7, − 105.9)**		−	Support		**− 87.0**	**(− 122.7, − 51.3)**		−	Support		**51.8**	**(14.6, 88.9)**	0	Conflict
	MVPA (min)	**51.2**	**(33.3, 69.0)**		+	Support		**50.7**	**(36.9, 64.6)**		+	Support		− 0.4	(− 12.4, 11.5)	0	Support
	Total steps	**5436.6**	**(4469.2, 6404.0)**		+	Support		**4951.0**	**(2213.8, 3562.3)**		+	Support		− 485.6	(− 1357.0, 385.8)	0	Support
	Sleep duration (min)	− 20.6	(− 43.9, 2.7)		+	Null		− 14.2	(− 34.6, 6.2)		+	Null		6.5	(− 11.6, 24.5)	0	Support
	Sleep onset	**− 52.9**	**(− 81.9, − 24.0)**		−	Support		**− 66.0**	**(− 84.3, − 47.6)**		−	Support		− 13.0	(− 40.4, 14.4)	0	Support
	I-I SD for sleep onset	**− 55.1**	**(− 91.0, − 19.1)**		−	Support		**− 45.3**	**(− 77.0, − 13.5)**		−	Support		9.8	(− 35.4, 55.0)	0	Support
	Sleep offset	**− 54.7**	**(− 122.6, − 68.3)**		−	Support		**− 90.5**	**(− 116.8, − 64.3)**		−	Support		4.9	(− 21.8, 31.7)	0	Support
	I-I SD for sleep offset	**− 71.5**	**(− 98.2, − 44.7)**		−	Support		**− 56.8**	**(− 83.6, − 30.0)**		−	Support		14.7	(− 21.8, 51.2)	0	Support
	Total screen time (min)	**− 44.0**	**(− 78.6, − 9.4)**		−	Support		**− 54.7**	**(− 91.8, − 17.7)**		−	Support		− 10.7	(− 44.6, 23.2)	0	Support
	Screen time after 20:00 h (min)	− 1.8	(− 23.3, 19.6)		−	Null		− 15.0	(− 40.0, 10.1)		−	Null		− 13.1	(− 39.6, 13.3)	0	Support
	Healthy foods/drinks	0.06	(− 0.19, 0.32)		+	Null		− 0.04	(− 0.25, 0.17)		+	Null		− 0.11	(− 0.36, 0.15)	0	Support
	Unhealthy foods/drinks	− 0.37	(− 0.92, 0.18)		−	Null		**− 0.52**	**(− 0.89, − 0.16)**		−	Support		− 0.16	(− 0.75, 0.44)	0	Support
21C (*n* = 57)	Sedentary (min)	**− 80.2**	**(− 112.3, − 48.2)**		−	Support		**− 86.4**	**(− 114.8, − 58.1)**		−	Support		− 6.2	(− 35.5, 23.1)	0	Support
	MVPA (min)	**15.0**	**(6.6, 23.4)**		+	Support		**16.1**	**(6.6, 23.4)**		+	Support		1.1	(− 5.0, 7.2)	0	Support
	Total steps	**2637.4**	**(1924.3, 3350.5)**		+	Support		**2888.1**	**(2213.8, 3562.3)**		+	Support		250.7	(− 284.4, 785.7)	0	Support
	Sleep duration (min)	**− 16.2**	**(− 31.5, − 0.9)**		+	Conflict		**− 17.3**	**(− 33.1, − 1.5)**		+	Conflict		− 1.1	(− 16.7, 14.5)	0	Support
	Sleep onset	**− 88.2**	**(− 115.0, − 61.3)**		−	Support		**− 88.4**	**(− 110.6, − 66.3)**		−	Support		− 0.3	(− 20.4, 19.9)	0	Support
	I-I SD for sleep onset	**− 53.5**	**(− 85.7, − 21.2)**		−	Support		**− 54.1**	**(− 85.2, − 23.0)**		−	Support		− 0.6	(− 37.4, 36.1)	0	Support
	Sleep offset	**− 104.8**	**(− 124.8, − 84.8)**		−	Support		**− 109.4**	**(− 129.8, − 89.1)**		−	Support		− 4.7	(− 23.4, 14.0)	0	Support
	I-I SD for sleep offset	**− 52.0**	**(− 94.2, − 9.9)**		−	Support		**− 37.7**	**(− 73.9, − 1.6)**		−	Support		14.3	(− 19.7, 48.3)	0	Support
	Total screen time (min)	**− 57.3**	**(− 92.0, − 22.6)**		−	Support		− 26.8	(− 55.8, 2.1)		−	Null		**30.4**	**(10.1, 50.8)**	0	Conflict
	Screen time after 20:00 h (min)	**− 22.9**	**(− 39.7, − 6.2)**		−	Support		− 4.6	(− 20.7, 11.4)		−	Null		**18.3**	**(6.5, 30.1)**	0	Conflict
	Healthy foods/drinks	− 0.17	(− 0.46, 0.11)		+	Null		0.13	(− 0.10, 0.35)		+	Null		**0.30**	**(0.10, 0.50)**	0	Conflict
	Unhealthy foods/drinks	**− 0.59**	**(− 1.06, − 0.12)**		−	Support		− 0.21	(− 0.62, 0.20)		−	Null		**0.38**	**(0.07, 0.69)**	0	Conflict
Control (*n* = 43)	Sedentary (min)													12.9	(− 49.0, 23.2)	0	Support
	MVPA (min)													2.0	(− 12.2, 8.2)	0	Support
	Total steps													26.0	(− 742.5, 690.5)	0	Support
	Sleep duration (min)													2.5	(− 16.0, 11.0)	0	Support
	Sleep onset													1.3	(− 15.4, 18.1)	0	Support
	I-I SD for sleep onset													− 26.7	(− 77.9, 24.5)	0	Support
	Sleep offset													− 4.1	(− 26.9, 18.7)	0	Support
	I-I SD for sleep offset													0.4	(− 42.0, 42.8)	0	Support
	Total screen time (min)													9.2	(− 41.0, 22.6)	0	Support
	Screen time after 20:00 h (min)													3.3	(− 17.5, 24.2)	0	Support
	Healthy foods/drinks													0.14	(− 0.31, 0.03)	0	Support
	Unhealthy foods/drinks													− 0.01	(− 0.39, 0.40)	0	Support

*Abbreviations*: *HSL* Healthy Summer Learners, *21C* 21st Century Program, *HR* hypothesized relationship, *HS* hypothesized support (i.e., did the relationship support the hypothesis), *I-I* intra-individual; estimates are based on multilevel mixed effects linear regressions with days nested within children, all estimates represent combined data from schools 1 and 2 (i.e., summer 2018 and 2019); bolded values indicated a statistically significant difference at *p* < 0.05. *Support* indicates statistically significant difference in the hypothesized direction or no statistically significant difference when no difference was hypothesized, *Null* indicates no statistically significant difference despite a hypothesis that the means should differ, and *Conflict* indicates statistically significant difference that is contrary to the hypothesized relationship. 0 indicates no significant difference

*Hypothesis 4. On days when they attend a program, children will engage in more beneficial levels of obesogenic behaviors compared to the weekend.*

Table 4 also presents the findings for hypothesis 4. In HSL, consistent with the hypothesis, sedentary time, MVPA, sleep onset and offset, sleep onset and offset variability, total screen time, and unhealthy foods/drinks were more favorable on days a child attended compared to weekend days. However, sleep duration, screen time after 20:00 h, and healthy foods/drinks were not statistically significantly different on days a child attended compared to weekend days. In 21C, consistent with the hypotheses, differences in sedentary time, MVPA, total steps, sleep onset and offset, and sleep onset and offset variability were more favorable on days a child attended compared to weekend days. However, total screen time, screen time after 20:00 h, and healthy and unhealthy foods/drinks were no different on days a child attended compared to weekend days, and, contrary to the hypothesis, sleep duration was more favorable on weekend days compared to days a child attended 21C.

*Hypothesis 5. On weekdays when children do not attend a program, all children (enrolled in a summer program and not enrolled) will engage in similar obesogenic behaviors compared to weekend days.*

Table 4 also presents the findings for hypothesis 5. In HSL, MVPA, total steps, total sleep time, sleep onset and offset, sleep onset and offset variability, total screen time, screen time after 20:00 h, healthy foods/drinks, and unhealthy foods/drinks were not statistically significantly different and supported the hypothesis. Contrary to the hypothesis, sedentary time was statistically significantly greater on weekdays a child did not attend a program compared to weekend days. In 21C, consistent with the hypothesis, sedentary time, MVPA, total steps, total sleep, sleep onset and offset, and sleep onset and offset variability were not statistically significantly different on weekdays that children did not attend 21C compared to weekend days. However, contrary to the hypothesis, total screen time, total screen time after 20:00 h, and healthy and unhealthy foods/drinks were statistically significantly different. In the control, all relationships supported the research hypothesis.

*Hypothesis 6. On days when children in HSL or 21C groups do not attend a summer program, they will engage in similar obesogenic behaviors compared to controls.*

Table 5 presents the findings for hypothesis 5. Consistent with the hypothesis, on weekdays that children did not attend HSL sedentary time, MVPA, sleep duration, sleep offset, sleep onset and offset variability, total screen time, screen time after 20:00 h, healthy foods/drinks, and unhealthy foods/drinks were not statistically significantly different from the control. However, contrary to the hypothesis, total steps and sleep onset were statistically significantly different between HSL and the control on weekdays children did not attend.

**Table 5:** Model estimated between program differences across weekdays not attended and weekend days

	HSL (*n* = 51) versus Control (*n* = 43)				21C (*n* = 57) versus Control (*n* = 43)				HSL (*n* = 51) versus 21C (*n* = 57)			
	Est	95% CI	HR	HS	Est	95% CI	HR	HS	Est	95% CI	HR	HS
**Hypothesis 6** **Weekday not attend**												
Sedentary (min)	4.1	(− 65.5, 73.6)	0	Support	− 58.0	(− 122.1, 6.1)	0	Support	62.1	(− 2.2, 126.5)	0	Support
MVPA (min)	3.6	(− 12.4, 19.7)	0	Support	7.7	(− 7.2, 22.5)	0	Support	− 4.0	(− 20.3, 12.3)	0	Support
Total steps	**1096.7**	**(59.4, 2134.0)**	0	Conflict	894.6	(− 198.1, 1987.3)	0	Support	202.1	(− 853.6, 1257.8)	0	Support
Sleep duration (min)	7.0	(− 21.0, 35.1)	0	Support	3.2	(− 19.2, 25.6)	0	Support	3.9	(− 20.7, 28.4)	0	Support
Sleep onset	**− 103.7**	**(− 144.4, − 62.9)**	0	Conflict	**− 51.5**	**(− 91.0, − 12.0)**	0	Conflict	**− 52.2**	**(− 93.2, − 11.2)**	0	Conflict
I-I SD for sleep onset	− 42.8	(− 98.4, 12.8)	0	Support	− 33.0	(− 83.8, 17.9)	0	Support	− 9.8	(− 52.4, 32.7)	0	Support
Sleep offset	− 54.7	(− 118.8, 9.4)	0	Support	− 45.7	(− 105.8, 14.4)	0	Support	− 9.0	(− 68.6, 50.6)	0	Support
I-I SD for sleep offset	− 27.5	(− 67.1, 12.0)	0	Support	− 19.6	(− 59.6, 20.5)	0	Support	− 7.9	(− 46.3, 30.4)	0	Support
Total screen time (min)	− 20.1	(− 67.6, 27.3)	0	Support	18.7	(− 25.7, 63.0)	0	Support	− 38.8	(− 79.1, 1.4)	0	Support
Screen time after 20:00 h (min)	− 19.2	(− 52.4, 14.1)	0	Support	2.0	(− 23.4, 27.4)	0	Support	− 21.1	(−50.2, 7.9)	0	Support
Healthy foods	0.01	(− 0.37, 0.40)	0	Support	0.10	(− 0.24, 0.43)	0	Support	− 0.08	(− 0.47, 0.30)	0	Support
Unhealthy foods	− 0.44	(− 1.15, 0.28)	0	Support	0.20	(− 0.53, 0.92)	0	Support	− 0.63	(− 1.42, 0.16)	0	Support
**Hypothesis 7** **Weekend day**												
Sedentary (min)	− 34.8	(− 108.1, 38.5)	0	Support	− 39.0	(− 111.3, 33.3)	0	Support	4.1	(− 60.0, 68.3)	0	Support
MVPA (min)	6.1	(− 8.1, 20.4)	0	Support	8.6	(− 6.9, 24.1)	0	Support	− 2.5	(− 19.6, 14.6)	0	Support
Total steps	**1608.3**	**(578.1, 2638.4)**	0	Conflict	669.9	(− 427.8, 1767.7)	0	Support	938.3	(− 269.3, 2146.0)	0	Support
Sleep duration (min)	3.1	(− 16.6, 22.7)	0	Support	6.8	(− 11.8, 25.3)	0	Support	− 3.7	(− 22.9, 15.5)	0	Support
Sleep onset	**− 92.0**	**(− 133.4, −50.6)**	0	Conflict	**− 52.6**	**(− 89.6, − 15.6)**	0	Conflict	**− 39.4**	**(− 74.3, − 4.6)**	0	Conflict
I-I SD for sleep onset	− 52.6	(− 105.5, 0.4)	0	Support	− 32.3	(− 82.8, 18.1)	0	Support	− 20.2	(− 59.1, 18.7)	0	Support
Sleep offset	− 55.5	(− 120.1, 9.1)	0	Support	− 36.9	(− 97.9, 24.1)	0	Support	− 18.6	(− 78.1, 40.9)	0	Support
I-I SD for sleep offset	**− 42.2**	**(− 81.7, − 2.7)**	0	Conflict	− 33.9	(− 68.2, 0.5)	0	Support	− 8.3	(− 40.6, 23.9)	0	Support
Total screen time (min)	− 0.3	(− 54.7, 54.2)	0	Support	− 2.6	(− 47.8, 42.6)	0	Support	2.3	(− 43.4, 48.1)	0	Support
Screen time after 20:00 h (min)	− 9.4	(− 55.3, 36.6)	0	Support	− 19.7	(− 50.7, 11.4)	0	Support	10.3	(− 28.2, 48.7)	0	Support
Healthy foods/drinks	0.26	(− 0.14, 0.66)	0	Support	− 0.06	(− 0.43, 0.30)	0	Support	0.32	(− 0.03, 0.67)	0	Support
Unhealthy foods/drinks	− 0.29	(− 0.92, 0.34)	0	Support	− 0.19	(− 0.81, 0.43)	0	Support	− 0.10	(− 0.81, 0.61)	0	Support

*Abbreviations*: *HSL* Healthy Summer Learners, *21C* 21st Century Program, *HR* hypothesized relationship, *HS* hypothesized support (i.e., did the relationship support the hypothesis), *I-I* intra-individual; estimates are based on multilevel mixed effects linear regressions with days nested within children, all estimates represent combined data from schools 1 and 2 (i.e., summer 2018 and 2019); bolded values indicated a statistically significant difference at *p* < 0.05. *Support* indicates statistically significant difference in the hypothesized direction or no statistically significant difference when no difference was hypothesized, *Null* indicates no statistically significant difference despite a hypothesis that the means should differ, and *Conflict* indicates a statistically significant difference that is contrary to the hypothesized relationship. 0 indicates no significant difference

When comparing 21C to the control and HSL to 21C, all relationships supported hypothesis 6 except sleep onset.

*Hypothesis 7. There will be no group differences in obesogenic behaviors on weekend days between HSL, 21C, and no program.*

Table 5 also presents hypothesis 7. When comparing weekend days between HSL to control, sedentary time, MVPA, sleep duration, sleep onset variability, sleep offset, total screen time, screen time after 20:00 h, and healthy and unhealthy foods/drinks supported the hypothesis. However, total steps, sleep onset, and sleep offset variability conflicted with the hypothesis. When comparing weekend days between 21C and control, sedentary, MVPA, total steps, sleep duration, sleep offset, sleep onset and offset variability, total screen time, screen time after 20:00 h, and healthy and unhealthy foods/drinks supported the hypothesis, while sleep onset conflicted with the hypothesis. When comparing weekend days between HSL and 21C sedentary time, MVPA, total steps, sleep duration, sleep offset, sleep onset and offset variability, total screen time, screen time after 20:00 h, and healthy and unhealthy foods/drinks supported the hypothesis, while sleep onset conflicted with the hypothesis.

Finally, Table 6 presents the total number of comparisons and the percent of comparisons that supported, conflicted, or were null when considering the hypotheses. Overall, 80.2% of the comparisons supported the hypotheses, 11.5% were null, and 8.3% conflicted.

**Table 6:** Comparisons classified as supporting, null, or conflicting the hypotheses

	Number	Percentage		Number	Percentage
**All hypotheses**			**Hypothesis 1**		
Total comparisons	192		Total comparisons	24	
Support	154	80.2	Support	16	66.7
Null	22	11.5	Null	8	33.3
Conflict	16	8.3	Conflict	0	0.0
**Hypothesis 2**			**Hypothesis 3**		
Total comparisons	12		Total comparisons	24	
Support	10	83.3	Support	18	75.0
Null	2	16.7	Null	5	20.8
Conflict	0	0.0	Conflict	1	4.2
**Hypothesis 4**			**Hypothesis 5**		
Total comparisons	24		Total comparisons	36	
Support	16	66.7	Support	31	86.1
Null	7	29.2	Conflict	5	13.9
Conflict	1	4.2			
**Hypothesis 6**			**Hypothesis 7**		
Total comparisons	36		Total comparisons	36	
Support	32	88.9	Support	31	86.1
Conflict	4	11.1	Conflict	5	13.9

*Support* indicates statistically significant difference in the hypothesized direction or no statistically significant difference when no difference was hypothesized, *Null* indicates no statistically significant difference despite a hypothesis means should differ, *Conflict* indicates a statistically significant difference that is contrary to the hypothesized direction or no statistically significant difference when a difference was hypothesized

## Discussion

This pilot study examined the impact of structured summer programming on the obesogenic behaviors of children. Overall, the results indicate that providing children with structured summer programming led to improvements in obesogenic behaviors during the summer months compared to children who were not provided access. Specifically, on days when children attended a program (i.e., HSL or 21C), they had more favorable patterns of obesogenic behaviors compared to controls for outcomes related to physical activity, sedentary behavior, sleep, screen time, and diet. This study identified the behavioral mechanisms impacted by HSL and will inform a future large-scale randomized trial.

Children who attended a program (i.e., HSL or 21C) showed significant improvements in obesogenic behaviors compared to their own behavior on days they did not attend the program (including both weekends and weekdays). On days children attended a structured summer program, they accumulated more steps, more overall MVPA, and less sedentary behavior compared to their own behavior on non-program days (either weekends or non-attended weekdays). These improvements were more pronounced in the HSL group, whereas improvements associated with program attendance in the domains of sleep, screen time, and diet were comparable across the two interventions. This was expected given that HSL was designed to provide additional opportunities for physical activity and healthy eating when compared to the 21C and neither HSL nor 21C explicitly targeted sleep or screen time.

This study also showed that on program nights children went to bed earlier and awoke earlier the next day, potentially driven by the early start time for summer programming. Similarly, children engaged in less screen time on program days. Together, these findings indicate that parents are more likely to enforce screen and bedtime rules in preparation for an early rise the next day. These findings are consistent with the literature that children report less screen time on school weekdays than the weekend [14], and evidence showing increased screen usage during summer months compared to the school year [15, 19]. Thus, the structure afforded by summer programming may have a beneficial impact on pre-bedtime screen time, which can positively shape sleep behaviors such as consistent bed/wake times and improve sleep quality [41].

Results concerning sleep duration warrant attention. Specifically, children obtained relatively less sleep on program days, a result consistent with past research exploring sleep duration on school vs. weekend days [42] or other school holidays [43]. Although a consistent link has been established between short sleep duration and risk for obesity in children [44], recent research regarding sleep variability (in both duration and timing) appears to indicate that consistency, in addition to duration, plays a role in obesogenic behavior patterns and ultimately obesity risk. Therefore, it is not clear whether the benefits of structure may outweigh the risks associated with shortened sleep.

Although we expected diet would be affected by summer program attendance, this relationship was inconsistent. The use of parental report and food frequency questionnaires may not be sufficiently sensitive to capture changes in children’s diet between structured and less structured days. Further, parents may not be aware of what children are eating on days that they attend a structured program. Although the current study aimed to balance precision with increased participant burden, a multiple pass 24-h dietary recall, the gold standard of free-living dietary assessment [45] may be necessary in future studies.

### Structured days

Overall, the results largely support the SDH with children engaging in more favorable obesogenic behaviors on days that they attended a structured summer program versus days they did not. Further, the finding that children displayed similar obesogenic behaviors on days that they did not attend a structured program further supports the SDH hypothesis. These findings demonstrate that increased access to structured summer programming may be an effective strategy to mitigate the obesogenic behaviors of children during the summer.

Structured summer programming offers children opportunities to engage in healthy behaviors and limits the opportunity to engage in unhealthy behavior. Moreover, consistent with the Theory of Expanded, Extended, and Enhanced opportunities, HSL increased children’s activity (MVPA, steps) and decreased sedentary time to a greater degree than the 21C [46]. This is likely because HSL provided 3 h of physical activity opportunities, while 21C provided 1 h. Thus, it appears that the SDH is a viable conceptual framework on which to base interventions targeting obesogenic behaviors in order to mitigate accelerated BMI gain during the summer.

### Strengths and limitations

This study has several strengths. First, this study was grounded in a theoretical framework: the SDH. Second, the study was based on set of closely related hypotheses. By testing a series of closely related hypotheses, causal inference is strengthened [38]. Third, this study extends existing evidence related to structured summer programming as an intervention strategy for the mitigation of increased engagement in obesogenic behaviors during summer. Third, it captured obesogenic behaviors over an extended period of time (12 weeks) and included objective measurements of activity and sleep. Lastly, the inclusion of a no contact control and academic-focused structured program (i.e., 21C) as control groups is a strength.

This study also has several limitations. First, this study used a relatively small sample size (*n* = 90), and while children were randomly assigned to HSL or no program, the schools assigned children to the 21C. The lack of random assignment to 21C could have led to unequal distribution of measured and unmeasured confounders between the intervention groups. Notably, the proportion of girls in 21C group is much lower than that in the HSL and no program groups. Second, screen time and diet data were collected via parent proxy-report, which may lead to potential bias in the estimates. Third, the use of a consumer wearable device could be considered a limitation. However, Fitbit has been shown to produce estimates of sleep and heartrate that have good agreement with polysomnography assessment of sleep and electrocardiography assessment of heartrate [21–23]. Fitbits were chosen for this study to allow for extended wear periods during the school year and summer. Additionally, the differential program lengths (HSL = 6 weeks, 21C = 4 weeks first summer, and 6 weeks second summer) may confound the observed findings and are acknowledged as a limitation of the study. Finally, the use of a food frequency questionnaire is another limitation as it may not be sensitive enough to capture changes in children’s diet. Moreover, parents may not have been aware of what their child ate on camp days or weekends, which constitutes a limitation on the survey’s internal validity.

## Conclusion

Providing children with structured programming during the “critical window” of summer may be a viable intervention strategy to reduce children’s engagement in obesogenic behaviors and potentially mitigate unhealthy BMI gain during the summer. Consistent with the SDH, children engaged in more healthful behaviors on days they attended a structured summer program. The findings of this study warrant future investigation into the impact of structured summer programming on children’s obesogenic behaviors. These results will be considered in concert with the academic and BMI *z*-score findings from the HSL pilot trial to determine if a full-scale trial is warranted.

## Supplementary information

**Additional file 1: Supplemental Table 1.** Behavioral data summer 2018 & 2019 for no program, Healthy Summer Learners and 21^st^ Century Learning by condition. **Supplemental Table 2.** Behavioral data summer 2018 for no program, Healthy Summer Learners and 21^st^ Century Learning by condition. **Supplemental Table 3.** Behavioral data summer 2019 for no program, Healthy Summer Learners and 21^st^ Century Learning by condition

## Data Availability

The datasets generated and/or analyzed during the current study are not publicly available due to institutional review board requirements but are available from the corresponding author on reasonable request.

## Abbreviations

HR: Hypothesized relationship
HS: Hypothesized support
HSL: Healthy Summer Learner’s program
21C: 21st Century Learning program
MVPA: Moderate-to-vigorous physical activity
SDH: Structured-days hypothesis
ST: Screen time
USDA: United States Department of Agriculture
