# Supplementary material for: The impact of summer programming on the obesogenic behaviors of children: behavioral outcomes from a quasi-experimental pilot trial
Source: Pilot Feasibility Stud. 2020 May 28;6:78. doi: 10.1186/s40814-020-00617-x (PMC7254707; doi:10.1186/s40814-020-00617-x)
Supplement: Supplementary file 1 — Additional file 1: Supplemental Table 1. Behavioral data summer 2018 & 2019 for no program, Healthy Summer Learners and 21st Century Learning by condition. Supplemental Table 2. Behavioral data summer 2018 for no program, Healthy Summer Learners and 21st Century Learning by condition. Supplemental Table 3. Behavioral data summer 2019 for no program, Healthy Summer Learners and 21st Century Learning by condition [file 40814_2020_617_MOESM1_ESM.docx]

| Supplemental Table 1. Behavioral data summer 2018 & 2019 for no program, Healthy Summer Learners and 21^st^ Century Learning by condition | | | | | | | | | | | | | | | | | |
| --- | --- | --- | --- | --- | --- | --- | --- | --- | --- | --- | --- | --- | --- | --- | --- | --- | --- |
|  | No Program | | | | | | | | | | | | | | | | |
|  | Attend Weekday | | | | |  | Not Attend Weekday | | | | |  | Weekend day | | | | |
|  | Mean | SD | Median | Interquartile Range | |  | Mean | SD | Median | Interquartile Range | |  | Mean | SD | Median | Interquartile Range | |
| Minutes Sedentary | - | - | - | - | - |  | 469.10 | (180.0) | 433.8 | (279.5, | 562.4) |  | 454.40 | (220.1) | 442.6 | (302.4, | 584.1) |
| Minutes MVPA | - | - | - | - | - |  | 58.20 | (31.7) | 37.4 | (16.3, | 71.5) |  | 53.90 | (27.4) | 39.2 | (17.4, | 83.1) |
| Total Steps | - | - | - | - | - |  | 7739.40 | (2145.2) | 7201.5 | (4812.0, | 9988.0) |  | 7772.30 | (2437.1) | 7166.0 | (5193.0, | 9774.0) |
| Minutes of Sleep Duration | - | - | - | - | - |  | 467.00 | (59.7) | 475.2 | (390.0, | 538.1) |  | 464.80 | (60.6) | 470.4 | (393.7, | 532.0) |
| Sleep Onset Time | - | - | - | - | - |  | 1:13:19 | (73.0) | 24:49:59 | (23:41:40, | 2:28:19) |  | 1:10:00 | (93.9) | 1.03 | (23:51:39, | 2:26:40) |
| I-I SD for Sleep Onset | - | - | - | - | - |  | 148.50 | (106.8) | 114.0 | (79.4, | 175.0) |  | 122.40 | (69.9) | 98.1 | (74.8, | 145.5) |
| Sleep Offset Time | - | - | - | - | - |  | 9:53:20 | (119.7) | 09:36:39 | (07:56:40, | 11:31:39) |  | 9:45:00 | (107.0) | 09:26:40 | (07:51:40, | 10:51:39) |
| I-I SD for Sleep Offset | - | - | - | - | - |  | 132.40 | (73.7) | 111.1 | (84.6, | 166.6) |  | 133.40 | (75.5) | 120.1 | (81.7, | 146.6) |
| Minutes Total Screen Time | - | - | - | - | - |  | 187.70 | (108.9) | 180.0 | (90.0, | 300.0) |  | 177.90 | (103.3) | 150.0 | (90.0, | 240.0) |
| Minutes Screen Time After 20:00h | - | - | - | - | - |  | 67.10 | (68.7) | 60.0 | (0.0, | 120.0) |  | 39.80 | (55.4) | 60.0 | (0.0, | 180.0) |
| Healthy Foods | - | - | - | - | - |  | 1.40 | (0.8) | 1.0 | (0.0, | 2.0) |  | 1.20 | (0.9) | 1.0 | (0.0, | 2.0) |
| Unhealthy Foods | - | - | - | - | - |  | 2.60 | (1.4) | 2.0 | (1.0, | 4.0) |  | 2.50 | (1.1) | 2.0 | (1.0, | 3.0) |
|  | Healthy Summer Learners | | | | | | | | | | | | | | | | |
|  | Mean | SD | Median | Interquartile Range | |  | Mean | SD | Median | Interquartile Range | |  | Mean | SD | Median | Interquartile Range | |
| Minutes Sedentary | 339.60 | (142.3) | 302.0 | (200.2, | 441.1) |  | 483.50 | (181.0) | 452.0 | (303.8, | 614.5) |  | 441.50 | (157.3) | 420.6 | (287.0, | 578.9) |
| Minutes MVPA | 107.80 | (67.9) | 93.1 | (52.2, | 146.1) |  | 57.10 | (54.6) | 39.2 | (14.8, | 82.9) |  | 58.90 | (44.3) | 38.9 | (16.4, | 75.8) |
| Total Steps | 14185.70 | (3735.6) | 14072.5 | (11211.0, | 16937.0) |  | 9136.10 | (3095.7) | 8619.0 | (5878.0, | 12283.0) |  | 9644.40 | (2716.8) | 9286.5 | (6320.0, | 12277.5) |
| Minutes of Sleep Duration | 447.30 | (42.7) | 450.9 | (411.5, | 484.3) |  | 476.40 | (55.1) | 480.1 | (434.1, | 531.3) |  | 466.70 | (60.5) | 468.7 | (416.1, | 515.7) |
| Sleep Onset Time | 22:31:40 | (117.8) | 22:28:19 | (21:46:40, | 23:13:19 |  | 23:19:59 | (76.9) | 23:15:00 | (22:16:40, | 0:33:19) |  | 23:36:39 | (106.0) | 23:21:39 | (22:21:39, | 0:23:20) |
| I-I SD for Sleep Onset | 47.10 | (28.8) | 39.0 | (29.4, | 51.7) |  | 103.00 | (81.7) | 76.7 | (35.9, | 132.4) |  | 92.60 | (73.5) | 75.7 | (46.3, | 112.5) |
| Sleep Offset Time | 6:35:00 | (200.0) | 06:33:19 | (06:06:40, | 06:56:40) |  | 8:11:40 | (114.0) | 07:56:40 | (06:43:20, | 09:28:20) |  | 8:09:59 | (190.0) | 08:03:19 | (06:45:00, | 09:01:39) |
| I-I SD for Sleep Offset | 29.00 | (13.1) | 29.8 | (21.6, | 38.5) |  | 101.70 | (61.8) | 92.4 | (63.8, | 118.0) |  | 86.40 | (65.0) | 69.2 | (44.4, | 100.2) |
| Minutes Total Screen Time | 107.80 | (96.6) | 90.0 | (30.0, | 180.0) |  | 166.10 | (81.4) | 150.0 | (90.0, | 210.0) |  | 171.50 | (142.4) | 150.0 | (90.0, | 210.0) |
| Minutes Screen Time After 20:00h | 40.20 | (94.6) | 30.0 | (0.0, | 60.0) |  | 55.50 | (136.2) | 30.0 | (0.0, | 60.0) |  | 28.10 | (157.9) | 30.0 | (0.0, | 60.0) |
| Healthy Foods | 1.60 | (0.9) | 2.0 | (1.0, | 3.0) |  | 1.60 | (0.9) | 2.0 | (1.0, | 3.0) |  | 1.80 | (0.9) | 2.0 | (1.0, | 3.0) |
| Unhealthy Foods | 1.90 | (1.4) | 2.0 | (1.0, | 4.0) |  | 2.40 | (1.5) | 2.0 | (1.0, | 3.0) |  | 2.30 | (2.1) | 2.0 | (1.0, | 3.0) |
|  | 21 Century Learning Center | | | | | | | | | | | | | | | | |
|  | Mean | SD | Median | Interquartile Range | |  | Mean | SD | Median | Interquartile Range | |  | Mean | SD | Median | Interquartile Range | |
| Minutes Sedentary | 349.70 | (135.9) | 316.8 | (223.3, | 442.6) |  | 418.40 | (168.9) | 389.3 | (262.4, | 534.5) |  | 435.90 | (215.0) | 403.7 | (274.2, | 562.2) |
| Minutes MVPA | 72.40 | (53.4) | 54.8 | (30.6, | 88.0) |  | 59.40 | (31.1) | 42.1 | (18.4, | 83.4) |  | 60.30 | (46.8) | 40.9 | (18.4, | 76.4) |
| Total Steps | 10890.40 | (2956.9) | 10415.0 | (8144.0, | 12709.0) |  | 9003.40 | (2995.7) | 8475.0 | (6071.5, | 11303.0) |  | 8710.60 | (8710.6) | 8043.0 | (5563.0, | 10878.0) |
| Minutes of Sleep Duration | 459.50 | (62.5) | 472.1 | (417.0, | 507.7) |  | 474.40 | (42.7) | 479.8 | (423.7, | 528.6) |  | 474.30 | (65.7) | 476.7 | (422.4, | 537.3) |
| Sleep Onset Time | 22:45:00 | (67.1) | 22:41:40 | (21:53:20, | 23:46:40 |  | 0:15:00 | (141.1) | 23:49:59 | (22:41:40, | 1:23:20) |  | 0:16:40 | (77.4) | 23:58:19 | (23:01:40, | 1:23:20) |
| I-I SD for Sleep Onset | 62.10 | (48.9) | 52.2 | (33.7, | 79.3) |  | 116.40 | (83.0) | 100.1 | (58.1, | 159.5) |  | 116.50 | (71.3) | 90.9 | (79.2, | 125.5) |
| Sleep Offset Time | 7:16:39 | (173.3) | 07:03:19 | (06:45:00, | 07:26:40) |  | 8:55:00 | (138.7) | 07:08:19 | (08:23:20, | 10:06:39) |  | 9:01:39 | (117.2) | 08:43:20 | (07:31:39, | 09:50:00) |
| I-I SD for Sleep Offset | 61.60 | (84.5) | 33.0 | (15.1, | 63.6) |  | 114.50 | (88.7) | 84.2 | (64.3, | 135.9) |  | 99.30 | (53.8) | 81.0 | (65.6, | 124.5) |
| Minutes Total Screen Time | 141.00 | (78.9) | 150.0 | (60.0, | 210.0) |  | 187.10 | (108.6) | 180.0 | (90.0, | 270.0) |  | 164.20 | (97.9) | 150.0 | (90.0, | 240.0) |
| Minutes Screen Time After 20:00h | 21.30 | (24.1) | 60.0 | (0.0, | 90.0) |  | 53.30 | (89.1) | 60.0 | (30.0, | 120.0) |  | 45.90 | (54.4) | 60.0 | (0.0, | 120.0) |
| Healthy Foods | 1.40 | (0.8) | 1.0 | (0.0, | 2.0) |  | 1.50 | (0.9) | 2.0 | (1.0, | 3.0) |  | 1.20 | (0.8) | 2.0 | (0.0, | 1.0) |
| Unhealthy Foods | 2.40 | (1.1) | 2.0 | (1.0, | 3.0) |  | 2.70 | (2.3) | 2.0 | (1.0, | 4.0) |  | 2.40 | (1.9) | 2.0 | (1.0, | 3.0) |
| Abbreviations: “SD” Standard deviation, “MVPA” Moderate-to-vigorous physical activity, “I-I” intra-individual | | | | | | | | | | | | | | | | | |

| Supplemental Table 2. Behavioral data summer 2018 for no program, Healthy Summer Learners and 21^st^ Century Learning by condition | | | | | | | | | | | | | | | | | |
| --- | --- | --- | --- | --- | --- | --- | --- | --- | --- | --- | --- | --- | --- | --- | --- | --- | --- |
|  | No Program | | | | | | | | | | | | | | | | |
|  | Attend Weekday | | | | |  | Not Attend Weekday | | | | |  | Weekend day | | | | |
|  | Mean | SD | Median | Interquartile Range | |  | Mean | SD | Median | Interquartile Range | |  | Mean | SD | Median | Interquartile Range | |
| Minutes Sedentary | - | - | - | - | - |  | 417.24 | (165.8) | 413.1 | (279.8, | 538.6) |  | 392.53 | (163.0) | 400.6 | (262.1, | 513.6) |
| Minutes MVPA | - | - | - | - | - |  | 58.46 | (57.4) | 37.7 | (17.4, | 82.5) |  | 52.82 | (50.7) | 38.4 | (15.8, | 71.5) |
| Total Steps | - | - | - | - | - |  | 7656.41 | (3411.5) | 7257.5 | (4923.0, | 9882.0) |  | 7687.00 | (4137.0) | 7270.0 | (5061.5, | 10030.5) |
| Minutes of Sleep Duration | - | - | - | - | - |  | 472.83 | (112.8) | 471.8 | (394.4, | 564.6) |  | 460.72 | (96.6) | 462.6 | (384.3, | 532.0) |
| Sleep Onset Time | - | - | - | - | - |  | 1:18:19 | (178.3) | 0.04 | (23:41:40, | 2:53:20) |  | 1:11:40 | (170.0) | 0.00 | (23:48:19, | 2:31:40) |
| I-I SD for Sleep Onset | - | - | - | - | - |  | 148.58 | (95.9) | 120.1 | (89.8, | 175.0) |  | 146.87 | (70.6) | 109.8 | (96.3, | 211.7) |
| Sleep Offset Time | - | - | - | - | - |  | 10:08:20 | (190.0) | 09:50:00 | (07:58:20, | 12:09:59) |  | 09:45:00 | (193.3) | 09:21:39 | (07:26:40, | 11:00:00) |
| I-I SD for Sleep Offset | - | - | - | - | - |  | 142.11 | (76.4) | 119.2 | (97.7, | 176.7) |  | 159.32 | (78.4) | 131.0 | (117.2, | 216.7) |
| Minutes Total Screen Time | - | - | - | - | - |  | 181.15 | (136.9) | 180.0 | (60.0, | 300.0) |  | 204.71 | (165.1) | 225.0 | (90.0, | 300.0) |
| Minutes Screen Time After 20:00h | - | - | - | - | - |  | 72.92 | (88.6) | 30.0 | (0.0, | 120.0) |  | 119.52 | (123.5) | 60.0 | (0.0, | 210.0) |
| Healthy Foods | - | - | - | - | - |  | 1.34 | (1.0) | 1.0 | (0.0, | 2.0) |  | 0.98 | (1.0) | 1.0 | (0.0, | 2.0) |
| Unhealthy Foods | - | - | - | - | - |  | 2.27 | (1.6) | 2.0 | (1.0, | 3.0) |  | 2.61 | (1.4) | 3.0 | (2.0, | 4.0) |
|  | Healthy Summer Learners | | | | | | | | | | | | | | | | |
|  | Mean | SD | Median | Interquartile Range | |  | Mean | SD | Median | Interquartile Range | |  | Mean | SD | Median | Interquartile Range | |
| Minutes Sedentary | 285.97 | (119.2) | 260.5 | (191.7, | 364.2) |  | 429.37 | (168.7) | 415.6 | (294.0, | 552.0) |  | 394.00 | (158.7) | 397.9 | (270.9, | 471.1) |
| Minutes MVPA | 102.71 | (56.0) | 96.3 | (56.0, | 138.2) |  | 47.62 | (43.8) | 36.2 | (14.5, | 68.0) |  | 56.14 | (56.5) | 37.4 | (14.8, | 72.8) |
| Total Steps | 15669.62 | (3451.1) | 15781.0 | (13282.0, | 17690.0) |  | 9877.46 | (4031.8) | 9745.5 | (6754.0, | 12943.0) |  | 10479.05 | (4246.9) | 9968.0 | (7093.0, | 12865.0) |
| Minutes of Sleep Duration | 446.53 | (69.8) | 446.3 | (402.4, | 484.3) |  | 455.32 | (79.0) | 463.4 | (407.7, | 498.9) |  | 458.92 | (93.3) | 458.1 | (402.8, | 506.2) |
| Sleep Onset Time | 22:33:19 | (68.4) | 22:28:19 | (21:49:59, | 23:13:19 |  | 23:16:40 | (157.4) | 23:18:19 | (22:25:00, | 24:26:40 |  | 23:28:19 | (115.4) | 23:15:00 | (22:18:19, | 0:33:19) |
| I-I SD for Sleep Onset | 57.71 | (35.1) | 49.8 | (30.6, | 74.7) |  | 118.56 | (85.1) | 100.6 | (66.7, | 150.4) |  | 101.57 | (63.2) | 92.0 | (66.2, | 116.1) |
| Sleep Offset Time | 06:31:39 | (42.4) | 06:38:19 | (06:04:59, | 06:58:20) |  | 08:06:39 | (148.1) | 07:38:19 | (06:48:19, | 09:06:39) |  | 08:01:40 | (153.2) | 07:36:40 | (06:31:39, | 08:48:19) |
| I-I SD for Sleep Offset | 29.45 | (11.4) | 30.1 | (24.7, | 37.1) |  | 116.17 | (73.6) | 98.8 | (58.2, | 158.0) |  | 94.48 | (67.1) | 75.0 | (61.5, | 95.5) |
| Minutes Total Screen Time | 106.92 | (89.6) | 120.0 | (0.0, | 180.0) |  | 107.61 | (101.8) | 120.0 | (45.0, | 120.0) |  | 143.40 | (135.1) | 120.0 | (90.0, | 150.0) |
| Minutes Screen Time After 20:00h | 38.29 | (40.3) | 30.0 | (0.0, | 60.0) |  | 45.88 | (78.6) | 30.0 | (0.0, | 60.0) |  | 76.58 | (106.6) | 30.0 | (0.0, | 120.0) |
| Healthy Foods | 1.58 | (0.9) | 2.0 | (1.0, | 3.0) |  | 1.80 | (0.9) | 2.0 | (1.0, | 3.0) |  | 1.68 | (0.9) | 2.0 | (1.0, | 3.0) |
| Unhealthy Foods | 1.60 | (1.1) | 2.0 | (1.0, | 3.0) |  | 2.20 | (1.3) | 2.0 | (1.0, | 3.0) |  | 2.45 | (1.2) | 3.0 | (1.0, | 4.0) |
|  | 21 Century Learning Center | | | | | | | | | | | | | | | | |
|  | Mean | SD | Median | Interquartile Range | |  | Mean | SD | Median | Interquartile Range | |  | Mean | SD | Median | Interquartile Range | |
| Minutes Sedentary | 345.06 | (160.1) | 314.3 | (222.3, | 438.1) |  | 374.43 | (170.7) | 357.2 | (240.9, | 484.1) |  | 401.13 | (173.3) | 385.6 | (274.2, | 527.1) |
| Minutes MVPA | 79.92 | (68.8) | 65.2 | (38.2, | 104.8) |  | 62.03 | (52.9) | 46.7 | (22.3, | 85.2) |  | 60.29 | (58.6) | 42.6 | (18.7, | 84.6) |
| Total Steps | 10984.13 | (5079.2) | 9999.0 | (7569.0, | 12736.0) |  | 9577.16 | (4281.5) | 9084.0 | (6740.5, | 12100.0) |  | 9274.16 | (5192.4) | 8704.5 | (5831.0, | 11054.0) |
| Minutes of Sleep Duration | 440.60 | (67.4) | 440.6 | (403.6, | 487.0) |  | 475.35 | (86.3) | 480.5 | (424.0, | 527.4) |  | 468.07 | (96.8) | 463.3 | (420.6, | 534.6) |
| Sleep Onset Time | 23:15:00 | (102.4) | 23:23:20 | (22:34:59, | 0:00:00) |  | 0:30:00 | (163.5) | 23:58:19 | (22:46:40, | 1:38:20) |  | 0:13:19 | (154.0) | 0.00 | (22:56:40, | 1:23:20) |
| I-I SD for Sleep Onset | 66.65 | (61.3) | 49.8 | (39.6, | 76.0) |  | 127.21 | (88.4) | 104.9 | (67.4, | 175.0) |  | 114.27 | (79.7) | 87.9 | (71.9, | 135.6) |
| Sleep Offset Time | 07:25:00 | (117.3) | 07:20:00 | (06:58:20, | 07:33:19) |  | 09:06:39 | (171.7) | 08:33:19 | (07:10:00, | 10:18:19) |  | 09:00:00 | (144.6) | 08:41:40 | (07:26:40, | 09:56:40) |
| I-I SD for Sleep Offset | 54.24 | (85.8) | 32.6 | (10.1, | 50.4) |  | 120.54 | (95.0) | 83.5 | (70.6, | 136.0) |  | 100.63 | (55.0) | 81.3 | (65.8, | 109.4) |
| Minutes Total Screen Time | 131.25 | (92.3) | 120.0 | (60.0, | 180.0) |  | 146.35 | (97.2) | 120.0 | (90.0, | 240.0) |  | 107.33 | (80.8) | 120.0 | (60.0, | 150.0) |
| Minutes Screen Time After 20:00h | 45.68 | (52.9) | 15.0 | (0.0, | 120.0) |  | 48.15 | (54.2) | 30.0 | (0.0, | 60.0) |  | 44.57 | (51.1) | 30.0 | (0.0, | 90.0) |
| Healthy Foods | 1.32 | (0.9) | 2.0 | (0.0, | 4.0) |  | 1.20 | (0.9) | 1.0 | (0.0, | 2.0) |  | 1.07 | (1.0) | 1.0 | (0.0, | 2.0) |
| Unhealthy Foods | 2.32 | (1.2) | 2.0 | (1.0, | 3.0) |  | 2.26 | (1.3) | 2.0 | (1.0, | 3.0) |  | 1.93 | (1.2) | 2.0 | (1.0, | 3.0) |
| Abbreviations: “SD” Standard deviation, “MVPA” Moderate-to-vigorous physical activity, “I-I” intra-individual | | | | | | | | | | | | | | | | | |

| Supplemental Table 3. Behavioral data summer 2019 for no program, Healthy Summer Learners and 21^st^ Century Learning by condition | | | | | | | | | | | | | | | | | |
| --- | --- | --- | --- | --- | --- | --- | --- | --- | --- | --- | --- | --- | --- | --- | --- | --- | --- |
|  | No Program | | | | | | | | | | | | | | | | |
|  | Attend Weekday | | | | |  | Not Attend Weekday | | | | |  | Weekend day | | | | |
|  | Mean | SD | Median | Interquartile Range | |  | Mean | SD | Median | Interquartile Range | |  | Mean | SD | Median | Interquartile Range | |
| Minutes Sedentary | - | - | - | - | - |  | 571.39 | (296.4) | 506.4 | (345.3, | 790.3) |  | 583.61 | (324.0) | 523.9 | (362.0, | 804.8) |
| Minutes MVPA | - | - | - | - | - |  | 57.75 | (49.9) | 45.1 | (17.5, | 85.2) |  | 56.35 | (57.7) | 36.1 | (17.5, | 73.4) |
| Total Steps | - | - | - | - | - |  | 7900.03 | (4454.4) | 7001.5 | (4645.0, | 10597.5) |  | 7813.16 | (3577.0) | 7071.0 | (5193.0, | 9768.0) |
| Minutes of Sleep Duration | - | - | - | - | - |  | 454.73 | (88.6) | 476.8 | (388.9, | 523.3) |  | 480.93 | (82.4) | 498.2 | (448.4, | 534.3) |
| Sleep Onset Time | - | - | - | - | - |  | 1:06:39 | (142.7) | 0.02 | (23:48:19, | 2:03:19) |  | 0:08:20 | (99.2) | 0.03 | 0 | 2:00:00) |
| I-I SD for Sleep Onset | - | - | - | - | - |  | 148.32 | (133.2) | 79.8 | (70.9, | 223.3) |  | 70.07 | (28.3) | 68.5 | (46.8, | 98.5) |
| Sleep Offset Time | - | - | - | - | - |  | 09:21:39 | (142.4) | 09:16:39 | (07:56:40, | 10:25:00) |  | 09:45:00 | (93.1) | 09:50:00 | (08:58:20, | 10:30:00) |
| I-I SD for Sleep Offset | - | - | - | - | - |  | 112.93 | (68.4) | 86.1 | (77.2, | 139.7) |  | 77.87 | (18.4) | 81.7 | (68.5, | 93.6) |
| Minutes Total Screen Time | - | - | - | - | - |  | 193.09 | (124.6) | 180.0 | (90.0, | 300.0) |  | 158.62 | (119.8) | 150.0 | (60.0, | 180.0) |
| Minutes Screen Time After 20:00h | - | - | - | - | - |  | 91.27 | (83.8) | 60.0 | (30.0, | 120.0) |  | 68.44 | (80.7) | 30.0 | (0.0, | 120.0) |
| Healthy Foods | - | - | - | - | - |  | 1.52 | (0.9) | 2.0 | (1.0, | 3.0) |  | 1.49 | (0.9) | 2.0 | (1.0, | 3.0) |
| Unhealthy Foods | - | - | - | - | - |  | 2.86 | (2.0) | 3.0 | (2.0, | 4.0) |  | 2.45 | (1.5) | 2.0 | (1.0, | 3.0) |
|  | Healthy Summer Learners | | | | | | | | | | | | | | | | |
|  | Mean | SD | Median | Interquartile Range | |  | Mean | SD | Median | Interquartile Range | |  | Mean | SD | Median | Interquartile Range | |
| Minutes Sedentary | 400.94 | (233.7) | 378.7 | (237.4, | 524.3) |  | 537.03 | (311.6) | 504.4 | (318.6, | 695.3) |  | 483.40 | (247.6) | 432.6 | (313.9, | 634.8) |
| Minutes MVPA | 113.62 | (91.3) | 91.8 | (45.9, | 155.9) |  | 66.56 | (75.0) | 44.8 | (16.1, | 100.3) |  | 61.39 | (64.2) | 39.5 | (18.0, | 84.6) |
| Total Steps | 12491.14 | (4969.5) | 12221.0 | (8922.0, | 14899.0) |  | 8402.51 | (4472.8) | 7832.5 | (5282.5, | 10690.0) |  | 8909.64 | (4224.6) | 8351.0 | (5952.0, | 11342.0) |
| Minutes of Sleep Duration | 448.52 | (53.4) | 454.7 | (422.6, | 486.1) |  | 497.53 | (86.4) | 494.0 | (458.5, | 553.3) |  | 478.63 | (71.4) | 491.3 | (440.1, | 523.8) |
| Sleep Onset Time | 22:28:19 | (90.3) | 22:26:40 | (21:41:40, | 23:13:19 |  | 23:25:00 | (121.3) | 23:10:00 | (22:11:40, | 12:36:39) |  | 23:46:40 | (119.0) | 23:43:19 | (22:26:40, | 0:41:40) |
| I-I SD for Sleep Onset | 34.05 | (9.2) | 34.5 | (28.2, | 38.7) |  | 84.25 | (77.4) | 60.5 | (25.5, | 130.0) |  | 82.05 | (86.1) | 59.7 | (36.1, | 95.2) |
| Sleep Offset Time | 06:41:40 | (115.3) | 06:31:39 | (06:08:19, | 06:56:40) |  | 08:16:40 | (134.3) | 08:23:20 | (06:30:00, | 09:53:20) |  | 08:20:00 | (122.0) | 08:20:00 | (07:18:19, | 09:13:20) |
| I-I SD for Sleep Offset | 28.45 | (15.5) | 27.9 | (20.0, | 39.9) |  | 84.25 | (40.7) | 78.4 | (70.8, | 100.7) |  | 76.95 | (64.1) | 52.2 | (31.1, | 117.9) |
| Minutes Total Screen Time | 108.55 | (105.1) | 90.0 | (30.0, | 120.0) |  | 194.68 | (117.2) | 180.0 | (120.0, | 270.0) |  | 184.02 | (134.7) | 180.0 | (120.0, | 225.0) |
| Minutes Screen Time After 20:00h | 50.14 | (95.8) | 30.0 | (0.0, | 45.0) |  | 60.41 | (76.9) | 30.0 | (0.0, | 90.0) |  | 58.98 | (109.5) | 30.0 | (0.0, | 60.0) |
| Healthy Foods | 1.74 | (1.0) | 2.0 | (1.0, | 3.0) |  | 1.49 | (0.9) | 2.0 | (1.0, | 3.0) |  | 1.82 | (1.0) | 2.0 | (1.0, | 3.0) |
| Unhealthy Foods | 2.13 | (1.8) | 2.0 | (1.0, | 4.0) |  | 2.43 | (1.9) | 2.0 | (1.0, | 4.0) |  | 2.25 | (2.0) | 2.0 | (1.0, | 3.0) |
|  | 21 Century Learning Center | | | | | | | | | | | | | | | | |
|  | Mean | SD | Median | Interquartile Range | |  | Mean | SD | Median | Interquartile Range | |  | Mean | SD | Median | Interquartile Range | |
| Minutes Sedentary | 352.77 | (191.7) | 318.7 | (227.5, | 443.0) |  | 490.31 | (229.3) | 449.5 | (336.0, | 609.3) |  | 473.33 | (247.3) | 442.1 | (275.7, | 602.6) |
| Minutes MVPA | 67.56 | (83.9) | 48.6 | (26.5, | 81.3) |  | 55.18 | (70.5) | 32.9 | (15.0, | 70.0) |  | 60.27 | (82.1) | 36.7 | (18.4, | 69.1) |
| Total Steps | 10829.18 | (3502.1) | 10712.0 | (8513.0, | 12652.0) |  | 8063.63 | (4192.2) | 7479.0 | (5459.0, | 10468.5) |  | 8105.54 | (4672.1) | 7436.0 | (5094.0, | 10650.0) |
| Minutes of Sleep Duration | 475.09 | (75.8) | 485.7 | (450.0, | 521.5) |  | 473.36 | (83.2) | 478.3 | (421.7, | 528.6) |  | 484.16 | (84.9) | 488.2 | (438.4, | 542.0) |
| Sleep Onset Time | 22:21:39 | (82.9) | 22:13:19 | (21:38:20, | 22:51:39 |  | 23:56:40 | (146.4) | 23:43:19 | (22:41:40, | 1:10:00) |  | 0:19:59 | (129.0) | 23:55:00 | (23:06:39, | 1:31:40) |
| I-I SD for Sleep Onset | 56.58 | (28.9) | 55.0 | (33.7, | 82.0) |  | 101.37 | (74.9) | 89.4 | (52.3, | 144.2) |  | 119.66 | (60.0) | 106.8 | (86.0, | 125.2) |
| Sleep Offset Time | 07:10:00 | (146.8) | 06:55:00 | (06:36:40, | 07:13:20) |  | 08:43:20 | (157.5) | 08:16:40 | (07:03:19, | 09:58:20) |  | 09:03:19 | (124.6) | 08:45:00 | (07:46:39, | 09:43:20) |
| I-I SD for Sleep Offset | 70.54 | (85.1) | 35.6 | (21.8, | 63.6) |  | 105.95 | (81.4) | 85.0 | (53.3, | 135.8) |  | 97.26 | (54.4) | 80.7 | (65.5, | 129.5) |
| Minutes Total Screen Time | 145.79 | (106.3) | 150.0 | (60.0, | 240.0) |  | 220.16 | (135.2) | 120.0 | (120.0, | 300.0) |  | 190.52 | (129.8) | 180.0 | (90.0, | 270.0) |
| Minutes Screen Time After 20:00h | 60.27 | (56.7) | 60.0 | (30.0, | 90.0) |  | 104.51 | (91.2) | 90.0 | (45.0, | 150.0) |  | 76.14 | (63.3) | 60.0 | (30.0, | 120.0) |
| Healthy Foods | 1.40 | (1.0) | 1.0 | (0.0, | 2.0) |  | 1.69 | (1.0) | 2.0 | (1.0, | 3.0) |  | 1.25 | (1.1) | 1.0 | (0.0, | 2.0) |
| Unhealthy Foods | 2.44 | (1.8) | 2.0 | (1.0, | 3.0) |  | 3.02 | (2.2) | 3.0 | (2.0, | 4.0) |  | 2.66 | (2.1) | 2.0 | (1.0, | 3.0) |
| Abbreviations: “SD” Standard deviation, “MVPA” Moderate-to-vigorous physical activity, “I-I” intra-individual | | | | | | | | | | | | | | | | | |
